# The genetic variations in the mitochondrial genomes of three Luciolinae fireflies

**DOI:** 10.1080/23802359.2020.1806126

**Published:** 2020-08-26

**Authors:** Quan Liu, Xinhua Fu

**Affiliations:** a Hubei Insect Resources Utilization and Sustainable Pest Management Key Laboratory, College of Plant Science and Technology, Huazhong Agricultural University, Wuhan, Hubei, China; b Firefly Conservation Research Centre, Wuhan, Hubei, China

**Keywords:** *Pygoluciola qingyu*, *Emeia pseudosauteri*, *Abscondita terminalis*, mitochondrial genome, variation

## Abstract

This paper studied the complete mitochondrial genomes of three fireflies, *Pygoluciola qingyu, Emeia pseudosauteri* and *Abscondita terminalis*. We discussed the variations in the mitochondrial genomes of samples of each firefly from different populations. The mitochondrial genomes of *Abs. terminalis* and *P. qingyu* are very stable among their different populations. The mitochondrial genome of *E. pseudosauteri* shows some variations among the different populations, especially in the COI sequence.

## Introduction

Lampyridae consists of nine subfamilies, 100 genera, and more than 2000 species. The taxonomy of Lampyridae has historically been based on morphological classification. As DNA barcode technology has matured, it has been used to identify insects and has shown good insect species identification capability in recent years in groups such as Coleoptera (Suzuki et al. 2002), thrips (Glover et al. 2010), and Trichoptera (Hogg et al. 2009).

The mitochondrial genome and 18S rDNA are the most conserved genes. They are not only very efficient for classification but are also essential for understanding the evolution of firefly insects. Therefore, many scientists have studied the mitochondrial genome and the nuclear rDNA of fireflies. In recent years, 44 firefly mitochondrial genomes have been sequenced. Twenty six of these are complete mitochondrial genomes, and the rest are partial mitochondrial genomes (Wang et al. 2017; Chen et al. 2019; Wang and Fu 2019; Zhang and Fu 2019). In addition, 89 rDNA coding regions (18S regions) of fireflies have been sequenced thus far (Chen et al. 2019).

*Emeia pseudosauteri* is a special species of firefly that is only found in China. Fu et al. described *Emeia* gen. nov. for a single species *pseudosauteri* transferred from *Curtos* (Fu et al. 2012). *P. qingyu* was first found by Fu and Ballantyne. They described the morphology of its males, females (bursa structure) and larvae. In addition, *P. qingyu* is the first recorded synchronously flashing firefly from mainland China (Fu and Ballantyne 2008). *Abscondita terminalis* was found by Olivier in 1883. It was divided into *Abscondita*, a new genus of fireflies from Southeast Asia (Ballantyne et al. 2013). The mitochondrial genomes and the 18S rDNA of these three kinds of firefly have been studied (Chen et al. 2019).

We collected 5 firefly samples (*P. qingyu*: one, *Abs. terminalis*: one and *E. pseudosauteri*: three) from Sichuan and Hubei Province. Here, we assembled mitogenomes from *P. qingyu*, *Abs. terminalis* and *E. pseudosauteri*. And we assembled 18srDNA from the three *E. pseudosauteri* samples. Combing the three firefly species with those previously deposited in GenBank and then comparing different populations among the same species, we discussed the differences of the mitochondrial genome in different populations of each species.

## Materials and methods

### Firefly specimens

#### DNA extraction and sequencing

Whole-body genomic DNA was extracted from each individual using the Aidlab Genomic DNA Extraction Kit (Aidlab Co., Beijing, China) following the manufacturer’s protocols (Table 1).

**Table 1.** Specimen collection and storage information.

Firefly species	Number	Collection information	Storage site of samples/reference
*Pygoluciola qingyu*	F9	Niujie township, Yiliang County, Yunnan Province	Chen et al. 2019
*Abscondita terminalis*	F11	Menglun township, Mengla County, Xishuangbanna Prefecture, Yunnan Province	
*Emeia pseudosauteri*	F15	Tiantaishan, Qionglai city, Sichuan Province	
*Pygoluciola qingyu*	F10	Jiugong Mountain, Tongshan County, Xianning City, Hubei Province	Natural History Museum, Huazhong Agricultural University, Wuhan City, Hubei Province, China
*Abscondita terminalis*	F12	Jiangxia District, Wuhan city, Hubei Province	
*Emeia pseudosauteri*	F16-A	Xiapu town, Tongshan County, Xianning city, Hubei Province	
*Emeia pseudosauteri*	F16-B	Lv Xin Garden, Leshan city, Sichuan Province	
*Emeia pseudosauteri*	F16-C	Gaojiayang town, Changyang County, Yichan city, Hubei Province	
*Vesta saturnalis*	F22	China, Yunnan: Xishuangbanna Prefecture, Mengla County, Menglun Township, Tropical Botanic Garden	Chen et al. 2019

#### Sequence processing and annotation

Primers were designed according to the mt genomic sequences of closely related species (and the 18S Primers were designed according to the genomic sequences of closely related species), and the long PCR (LA-PCR) amplification was performed using the LA Taq polymerase. The PCR conditions were as follows: initial denaturation 94 °C for 2 min, then 35 cycles of denaturation at 94 °C for 30 s, annealing at 55 °C for 30 s, and extension at 72 °C for 1 min/kb, followed by the final extension at 72 °C for 10 min. The total volume for PCR and LA-PCR was 50 μl, of which Takara LATaq (5 U/μl) was 0.5 μl, 10 × LATaq Buffer II (Mg2+) was 5 μl, dNTP mixture (2.5 mM) was 8 μl, template was 60 ng, and the total volume was then made up with distilled water. The final concentration of the forward and reverse primers was 0.2 ∼ 1.0 μM, and that of MgCl2 was 2.0 mM. The PCR products were sequenced directly, or if needed first cloned into a pMD18-T vector (Takara, JAP) and then sequenced, by the dideoxynucleotide procedure, using an ABI 3730 automatic sequencer (Sanger sequencing) using the same set of primers.

After quality-proofing of the obtained fragments, the complete mt genome sequence was assembled manually using DNAstar v7.1 software (Burland 2000). Mt genome was annotated roughly following the procedure described before (Zou et al., 2017; Zhang et al., 2018b). First, raw mitogenomic sequences were imported into MITOS web servers to determine the approximate boundaries of genes. Exact positions of protein-coding genes (PCGs) were found by searching for ORFs (employing genetic code 5, the invertebrate mitochondrion). All tRNAs were identified using ARWEN, DOGMA and MITOS.

#### Morphological identification

We identified the morphological characteristics and dissected genital organs of male fireflies of each species by using a microscope. Then, the features were compared with previously published classification articles about the three kinds of fireflies.

#### Phylogenetic analyses

To reconstruct the phylogenetic relationship among the species, the COI sequences of 22 firefly species, one Cantharidae and one Tenebrionidae were obtained from the GenBank database (Table 2). These sequences were divided into 5 subfamilies. The sequences of *Tribolium castaneum* (F20, NC003081) and *Chauliognathus opacus* (F21, FJ613418) were used as outgroups. The COI sequences from the 24 species were used in phylogenetic analysis, which was performed using maximum likelihood (ML) method with the MEGA version 7.0 program.

**Table2.** The COI sequences of 24 species were obtained from the GenBank database.

Number	Subfamily	Species	Accession No.	Cite References
F1	Luciolinae	*Luciola substriata*	KP313820	Mu et al. 2015
F2		*Asymmetricata circumdata*	KX229747	Luan and Fu 2016
F3		*Pteroptyx maipo*	MF686051	Fan and Fu 2017
F4		*Aquatica leii*	KF667531	Jiao et al. 2015
F5		*Aquatica ficta*	KX758085	Wang et al. 2017
F6		*Aquatica wuhana*	KX758086	Wang et al. 2017
F7		*Aquatica lateralis*	LC306678	Maeda et al. 2017
F8		*Luciola cruciata*	AB849456	Amano et al 2013
F9		*Pygoluciola qingyu*	MK292093	Chen et al. 2019
F10		*Pygoluciola qingyu*	In this study	Tongshan County
F11		*Abscondita terminalis*	MK292092	Chen et al. 2019
F12		*Abscondita terminalis*	In this study	Wuhan city
F13		*Abscondita chinensis*	MK122952	Wang and Fu 2019
F14		*Abscondita anceyi*	MH020192	Hu and Fu 2018
F15		*Emeia pseudosauteri*	MK292112	Chen et al. 2019
F16-A		*Emeia pseudosauteri*	In this study	Tongshan County
F16-B		*Emeia pseudosauteri*	In this study	Leshan City
F16-C		*Emeia pseudosauteri*	In this study	Changyang County
F17		*Curtos costipennis*	MK609965	Zhang and Fu 2019
F18	Lampyrinae	*Pyrocoelia rufa*	AF452048	Bae et al 2004
F19		*Diaphanes citrinus*	MK292103	Yang and Fu 2019
F20	Tribolium	*Tribolium castaneum*	NC003081	Friedrich and Muqim 2003
F21	Chauliognathus	*Chauliognathus opacus*	FJ613418	Sheffield et al. 2009
F22	Amydetinae	*Vesta saturnalis*	MK292111	Chen et al. 2019

#### BLAST analyses

We identified the similarities and differences of mitochondrial DNA among the F11 and F12, the F9 and F10 by using MegaBLAST.

#### Multiple sequences alignment

We compared the 18sDNA sequences of the F-16A, F-16B and F-16C by using Clustal W with the MEGA version 7.0 program.

We compared the 18sDNA sequences of F15, F16 and F22 by using Clustal W with the MEGA version 7.0 program. The 18sDNA sequences of F15 and F22 were downloaded from GenBank.

We compared the MtDNA sequences of the F-16A, F15 and F22 by using Clustal W with the MEGA version 7.0 program.

## Results

### Species identification

By analyzing these morphology features, we confirmed that samples F16-A, F16-B, F16-C were *E. pseudosauteri,* F10 was *P. qingyu,* and F12 was *Abs. terminalis.* To further confirm the identification of the three F16 samples, we analyzed the 18S rDNA sequences of the three samples by Clustal W, and the result showed that they are *E. pseudosauteri.*

### Basic description of the sequences

The complete mitochondrial genome sequences of the three firefly species are similar to each other. As Metazoa, each of the sequences contains 13 protein-coding genes, 22 transfer RNA genes, 2 ribosomal RNA genes, and a non-coding AT-rich region, which together represent a typical insect mitochondrial genome (Wolstenholme 1992). The open frames of the 13 protein-coding genes were inferred from three other fireflies: *Aquatica leii*, *Luciola substriata*, and *Pyrocoelia rufa* (Lee et al. 2003; Jiao et al. 2015; Mu et al. 2015). Detailed information on the complete mitochondrial genome of the three kinds of firefly is provided in Table 3.

**Table 3.** Basic description of the complete mitochondrial genomes of the three species.

Species	*Pygoluciola qingyu* (F10)	*Abscondita terminalis* (F12)	*Emeia pseudosauteri* (F16)
Base length	17,332 bp	16,398 bp	16,956 bp
Base composition			
A	45.59%	41.19%	44.21%
C	11.66%	11.95%	11.46%
G	7.96%	8.24%	8.32%
T	34.71%	35.62%	36.00%
Initiation codon			
ATT	COI, D5, ND3, ATP8	COI, ND3, ND4L	COI
ATA	ND6, ND2	ND2, ATP8, ND5, ND6, ND1	ND2, ND5, ND3, ND6, ATP8
ATG	COII, OIII, ND4, ND4L, ATP6, CTYB	COII, COIII, ND4, ATP6, CTYB	COII, COIII, ND4, ND4 L, ATP6, CTYB, ND1
TTG	ND1		
Terminal codon			
Single T	COII, ND5, ND4, COI	COII, COIII, ND5, ND4, COI	COII, COIII, ND5, ND4, COI
TAA	ND2, ND6, ATP8, ATP6, ND4L	ND2, ND6, ATP8, ATP6, ND4L, D4L	ND2, ND6, ATP8, ATP6, ND4L
TAG	ND1, ND3, CTYB	ND1, CTYB	ND1, ND3, CTYB
AGA	COIII		COIII
GenBank accession No.	MN688374	MN722653	MN722654

### Results of phylogenetic analysis based on COI sequences

**Figure 1.**
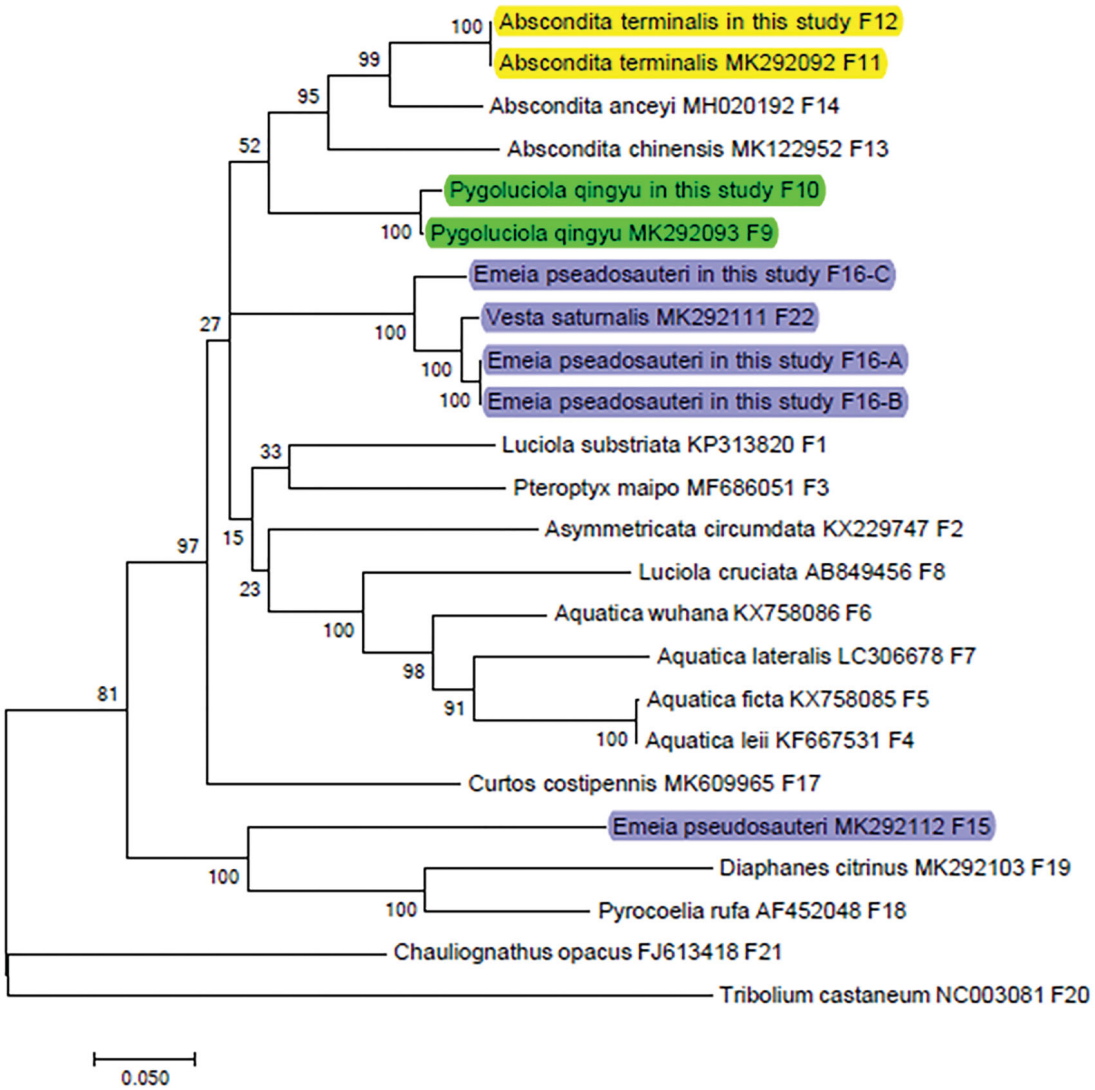
Molecular phylogenetic analysis by maximum likelihood method.

Based on Figure 1, we can draw the following conclusions: first, the COI sequences of F11 and F12, F9 and F10 are exactly the same. Then, the sequences of F16-A, F16-B are the same. The COI sequence of F22 and F16-C is slightly far from those of F16-A and F16-B. Finally, the genetic distance between the COI sequence of F15 and the other four COI sequences of F16 and F22 is very far.

### BLAST results

According to the BLAST results for the mitochondrial genome sequences, we can draw some conclusions as follows: first, the sequences of F11 and F12 are almost homologous, and their percent identity is 99.52%. Second, the sequences of F9 and F10 are very similar, and their percent identity is 98.43%. The differences are that the first 330 bases of F9 do not match those of F10, and the end of the F9 sequence lacks 919 bases.

### The results of multiple sequences alignment

According the multiple sequences alignment, the 18sDNA sequence of F22 is the same with F16, but the 18sDNA sequence of F15 has big difference with the F16. The MtDNA sequence of F16-A and F22 are almost the same, but they have big difference with the F15 (Tables 4 and 5).

**Table 4.** Estimates of evolutionary divergence between 18sDNA sequences.

Species	1	2	3	4	5
*Emeia pseadosauteri* in this study F16-A					
*Emeia pseadosauteri* in this study F16-B	0.000				
*Emeia pseadosauteri* in this study F16-C	0.000	0.000			
*Vesta saturnalis* MK292083 F22	0.000	0.000	0.000		
*Emeia pseudosauteri* MK292085 F15	0.582	0.582	0.582	0.582	

**Table 5.** Estimates of evolutionary divergence between MtDNA sequences.

Species	1	2	3
*Emeia pseadosauteri* in this study F16-A			
*Vesta saturnalis* MK292083 F22	0.022		
*Emeia pseudosauteri* MK292085 F15	0.234	0.234	

## Discussion

*Abscondita terminalis* is widely distributed in China, and it can survive in diverse habitat environments. To adapt to these diverse environments, the different geographical populations of *Abs. terminalis* may show some variations. We isolated two geographical populations that were about a thousand kilometers apart to compare the variations in their mitochondrial genomes. The results revealed that the genetic distance between the two populations is very small. That means that the mitochondrial genome does not vary among different geographical populations and that the heredity of this species is very stable.

*Pygoluciola qingyu* is also widely distributed in China. In addition, the two samples (F9 and F10) were gathered from more than a kilometer apart. The variation analysis of the two populations suggests that the mitochondrial genome sequence of *P. qingyu* is very stable.

The F9 sequence is a partial mitochondrial genome sequence that was downloaded from Genebank, while F10 is a complete mitochondrial genome sequence that we had sequenced. Although the two sequences are very similar, they are still show important differences. The F9 lost the control region of a noncoding region that contains a repetitive sequence. Scientists have shown that any sequence of the mitochondrial genome is very important, especially the control region in noncoding sequences and the coding regions. In addition, any missing sequence will cause coding confusion and functional changes.

From the analyses of the mitochondrial genome sequences and 18S rDNA sequences of the three firefly samples (F15, F16-A, and F22) as well as the results for the two species of fireflies (*E. pseudosauteri* and *V. saturnalis)* in the paper published by Xing Chen et al. (Chen et al. 2019), we conclude that the mitochondrial genome sequences and 18 s rDNA sequences of the two fireflies should be interchanged.

*Emeia pseudosauteri* is only distributed in Sichuan Province and Hubei Province, where there are many mountains, causing habitat isolation. The results above show that the COI sequence of F16-C is very different from the COI sequences of the other two populations, F16-A and F16-B. This means that the mitochondrial genome sequence of *E. pseudosauteri* shows great variation among different geographical populations.

The above discussion suggests that classification based on the mitochondrial genome is not very accurate and that human factors causing upload errors in Genebank may lead to incorrect identification. Scientists should combine morphological classification and DNA barcoding to classify firefly species in order to make the classification more accurate.

The above discussions reveal that the COI sequences in different populations have great variation. It means that the mtDNA may have variation among different geographical populations. In this paper we only sequenced the differences of COI sequences of *E. pseudosauteri*. We did not discuss the differences of complete MtDNA sequences of *E. pseudosauteri* in details, especially the differences of D-loop and AT-reach region of MtDNA sequences of *E. pseudosauteri* in different geographical populations.
